# Validation of a proposed WOMAC short form for patients with hip osteoarthritis

**DOI:** 10.1186/1477-7525-9-75

**Published:** 2011-09-21

**Authors:** Amaia Bilbao, José M Quintana, Antonio Escobar, Carlota Las Hayas, Miren Orive

**Affiliations:** 1 Basque Foundation for Health Innovation and Research (BIOEF)-CIBER Epidemiología y Salud Pública (CIBERESP), Sondika, Bizkaia, Spain; 2 Research Unit, 'Galdakao-Usansolo' Hospital-CIBER Epidemiología y Salud Pública (CIBERESP), Galdakao, Bizkaia, Spain; 3 Research Unit, 'Basurto' Hospital-CIBER Epidemiología y Salud Pública (CIBERESP), Bilbao, Bizkaia, Spain

**Keywords:** WOMAC, Short form, Hip replacement, Reliability, Validity, Responsiveness, Rasch analysis

## Abstract

**Background:**

The aims of this study were to propose a Spanish Western Ontario and McMaster Universities Osteoarthritis Index (WOMAC) short form based on previously shortened versions and to study its validity, reliability, and responsiveness for patients with hip osteoarthritis undergoing total hip replacement (THR).

**Methods:**

Prospective observational study of two independent cohorts (788 and 445 patients, respectively). Patients completed the WOMAC and the Short Form (SF)-36 questionnaires before THR and 6 months afterward. Patients received the questionnaires by mailing, and two reminder letters were sent to patients who had not replied the questionnaire. Based on two studies from the literature, we selected the two shortened domains, the pain domain composed of three items and the function domain composed of eight items. Thus, we proposed an 11-items WOMAC short form. A complete validation process was performed, including confirmatory factor analysis (CFA) and Rasch analysis, and a study of reliability, responsiveness, and agreement measured by the Bland-Altman approach.

**Results:**

The mean age was about 69 years and about 49% were women. CFA analyses confirmed the two-factor model. The pain and function domains fit the Rasch model. Stability was supported with similar results in both cohorts. Cronbach's alpha coefficients were high, 0.74 and 0.88. The highest correlations in convergent validity were found with the bodily pain and physical function SF-36 domains. Significant differences were found according to different pain and function severity scales, supporting known-groups validity. Responsiveness parameters showed large changes (effect sizes, 2.11 and 2.29). Agreement between the WOMAC long and short forms was adequate.

**Conclusions:**

Since short questionnaires result in improved patient compliance and response rates, it is very useful to have a shortened WOMAC version with the same good psychometric properties as the original version. The Spanish WOMAC short form is valid, reliable, and responsive for patients undergoing THR, and most importantly, the first WOMAC short version proposed in Spanish. Because of its simplicity and ease of application, the short form is a good alternative to the original WOMAC questionnaire and it would further enhance its acceptability and usefulness in clinical research, clinical trials, and in routine practice within the orthopaedic community.

## Background

The disease-specific questionnaire, Western Ontario and McMaster Universities Osteoarthritis Index (WOMAC), is the most widely used instrument to evaluate symptomatology and function in patients with hip or knee osteoarthritis (OA) [1-5]. The measure was developed to evaluate clinically important, patient-relevant changes in health status resulting from treatment interventions [6]. The WOMAC, which is self-administered and covers three dimensions: pain (5 items), stiffness (2 items), and physical function (17 items), is reliable, valid, and sensitive to changes in the health status of patients with hip or knee OA [1,7-10].

A major uses of health measurement scales is to detect health status changes over time, and a priority may be efficiency, i.e., responses achieved using the shortest possible questionnaire [11,12]. A shorter version would further enhance its applicability in epidemiologic studies, clinical trials, and daily clinical practice [13], since short questionnaires result in improved patient compliance and response rates and are thought to improve the quality of the response [14,15]. Traditionally, one of the major disadvantages of self-administered questionnaires has been the low response rate, which greatly affects the study validity [15,16], but it has been shown that shorter version of the questionnaires would significantly increase the response rate [15]. In addition, several studies have reported that the WOMAC function scale is redundant and suggested that the scale should be shortened by omitting the repetitious items [17,18]. Therefore, it would be very useful to have a shortened WOMAC version in Spanish, which retains the same good psychometric properties of the original version.

The WOMAC questionnaire has been shortened recently [11,19-21]. Some have been shortened using statistical approaches [19,20], and others by considering the perspective of patients and rheumatologists [11,21]. The stiffness domain of the WOMAC is largely redundant and is often excluded from the questionnaire [21]. Therefore, some authors have centred their studies on shortening the function domain [11,21], while others have shortened the pain and function domains [19,20], but these shortened domains have not been validated as a whole shortened WOMAC version, checking the existence of two underlying domains. Since the shortened scale is essentially a component of the fully shortened version, the subjacent structure of the reduced version should be analyzed.

The goal of the current study was to propose a shortened Spanish WOMAC version based on previously shortened versions and to evaluate the validity, reliability, and responsiveness of this shortened questionnaire for patients with hip OA, combining classical and modern statistical techniques, such as Rasch analysis.

## Methods

### Study population

The current study included data from two prospective cohorts recruited independently from various public teaching hospitals. Consecutive patients who underwent total hip replacement (THR) between March 1999 and March 2000, and between September 2003 and September 2004, were eligible for the study and included in cohort 1 and 2, respectively. In both cohorts, patients with main diagnosis different to hip or knee osteoarthritis (OA), or with a malignant pathology or other organic or psychiatric condition that prevented participation, or with failure to undergo surgical intervention were excluded. Each hospital's ethics review board approved the study.

### Measurements

The data collection and methodology for both cohorts were the same. All patients on the waiting list for a THR were mailed to their home address a letter that described the study and requested voluntary participation. The WOMAC [1], short Form (SF)-36 [22] questionnaires, and additional questions regarding the level of pain and function, which we will refer to as the categorical scales, were included in the mailing. The structure of those variables has been described previously [23], and they classified patients as having minor, moderate, and severe pain or function. Therefore, patients completed the questionnaires at home, and they returned them by mail. A reminder letter was sent to patients who had not replied after 15 days. The patients who still had not responded after another 15 days received the questionnaire again and were contacted by telephone to ask them about the reasons of their non response. Six months after the intervention, patients received the same questionnaires and the follow up for those not responding was as described previously. Sociodemographic and clinical data also were collected.

The SF-36 is a generic questionnaire on health-related quality of life [22] that has 36 items and covers eight domains (physical function, physical role, bodily pain, general health, vitality, social function, emotional role, and mental health) and two summary scales on physical and mental health. The scores for the SF-36 domains range from 0 to 100, with higher scores indicating better health status. The SF-36 has been translated into Spanish and validated in Spanish populations [24].

The WOMAC is a health status instrument specific for patients with hip or knee OA [1]. It has a multidimensional scale comprising 24 items grouped into three dimensions: pain (5 items), stiffness (2 items), and physical function (17 items). We used the Likert version of the WOMAC with five response levels for each item, representing different degrees of intensity that were scored from 0 (none) to 4 (extreme). The WOMAC has been translated into Spanish and validated in Spain [8-10].

After a thorough review of the literature and existing shortened WOMAC versions, we derived the WOMAC short form (WOMAC-SF) from the original WOMAC version to evaluate pain and function in patients with hip OA. The WOMAC pain short form was selected from a previously shortened version using Rasch analysis [19], which included items 1, 2, and 4 of the long form. The function short form included items 1, 2, 3, 6, 7, 8, 9, and 15 of the long form, selected from a previous study based on patients' and experts' opinions [11]. Some psychometric properties of the function short form have been investigated previously [25]. Therefore, the WOMAC-SF that we proposed has 11 items grouped into two dimensions: pain (3 items) and function (8 items). The final scores for the long and short WOMAC versions were determined by adding the aggregate scores for pain and function separately, and standardizing them to a range of values from 0 to 100, with 0 representing the best health status possible and 100 the worst.

### Statistical analysis

The unit of the study was the patient. In cases in which a patient underwent two interventions during the recruitment period, we selected the first intervention performed.

To describe the samples, we used means and standard deviations (SDs), frequencies, and percentages. We compared sociodemographic and clinical data and WOMAC domains at baseline between the cohorts. Chi-square or Fisher's exact tests were performed to compare categorical variables, and the t-test or the Wilcoxon nonparametric test was used to compare continuous variables.

Cohort 1 was used to study all the psychometric properties performed to validate the Spanish 11-item WOMAC-SF. With the aim of studying the stability of items performance across different samples to give more evidence of validity, analyses concerning the construct validity were replicated in cohort 2.

#### Construct validity

We studied the construct validity by means of confirmatory factor analysis (CFA) to investigate the hypothesis that the 11 items on the questionnaire addressed two factors, pain and function. Different fit indexes were evaluated [26-29]: the root mean square error of approximation (RMSEA), for which a value below 0.08 was considered acceptable; and the non-normed fit index (NNFI) and comparative fit index (CFI), both of which had to exceed 0.90 to be satisfactory. We also examined factor loadings, and those 0.40 or higher were considered acceptable. We performed the CFA in both cohorts to study the stability of the subjacent structure of the questionnaire.

We applied the Rasch method to the WOMAC pain and function short forms separately to ensure that the scales were unidimensional [17,30], a fundamental requirement of construct validity [31]. We assessed unidimensionality by means of infit and outfit statistics, with values between 0.7 and 1.3 indicating a good fit [32], and through a principal components analysis (PCA) of the residuals extracted from the Rasch model [19,20]. Unidimensionality was considered violated if, in addition to the first factors, other factors had eigenvalues exceeding 3 [33]. We evaluated the ability of the WOMAC-SF to define a distinct hierarchy of items along the measured variable by means of an item separation index [30]. A value of 2.0 or greater for this statistic is comparable to reliability of 0.80 and is acceptable. To detect the presence of differential item functioning (DIF), which occurs when different groups within the sample respond in a different manner to an individual item [34], we compared the different levels of the trait by gender. A Welch *t *statistically significant at *P*< 0.05, and a difference in difficulty of at least 0.5 logit was considered as noticeable DIF [33]. We performed Rasch analyses in both cohorts to study the stability of the item logits and item order across the different samples.

#### Reliability

We assessed reliability using Cronbach's alpha coefficient [35]. A coefficient over 0.70 was considered acceptable [36].

#### Convergent and discriminant validity

We assessed convergent and discriminant validity by analysing the relationship between the WOMAC-SF domains and the SF-36 domains with the Spearman correlation coefficient. We established that correlations between the WOMAC-SF domains and the other measures must be lower than the internal consistency of the WOMAC-SF scales [37]. We also hypothesized that the correlation between the WOMAC short pain scale and the bodily pain domain of the SF-36 and between the WOMAC short function scale and the physical function SF-36 domain would be higher than with the other domains.

#### Known-groups validity

We examined known-groups validation by comparing the WOMAC pain and function short scales among the different groups according to pain and function categorical scales [23]. We hypothesized that the more severe the patient's pain or function level, the higher their WOMAC pain and function short scores would be. Analysis of variance using the Scheffe test for multiple comparisons or the non-parametric Kruskal-Wallis test was performed for the analysis.

#### Responsiveness

We compared principal characteristics between patients who responded to the follow-up and those who did not. Means and SDs were calculated for the WOMAC-SF scales at baseline and 6 months after surgery. We used a paired t-test for the comparison before and after the intervention. Ceiling and floor effects at baseline and 6 months after surgery were examined to evaluate the discriminatory ability of the scales.

To measure the responsiveness of the WOMAC-SF, we used the standardized effect size (SES), defined as the mean change score divided by the SD of the baseline scores, and standardized response mean (SRM), defined as the mean change score divided by the SD of the change scores [38]. Cohen's benchmarks were used to classify the magnitude of the effect sizes [39].

#### Agreement between the long and the short womac forms

We evaluated the correlations between the pain and function long and short scales at baseline, 6 months after intervention, and for changes in scores by Spearman's correlation coefficient. Agreement between the WOMAC long and short scales was examined by the Bland-Altman approach [40], which is useful for searching for any systematic bias, assessing random error, and revealing whether the difference between the scores depends on the level of the scores [25].

All statistical analyses were performed with SAS for Windows statistical software, version 9.1 (SAS Institute, Inc., Cary, NC), except the Rasch analysis for which we used Winsteps version 3.69.1.4 software (John M. Linacre, Chicago).

## Results

During the recruitment period, we included 788 and 445 patients in the first and second cohorts respectively, who underwent a THR, fulfilled selection criteria, and accepted to participate. Of these, 590 (74.87%) and 339 (76.18%), respectively, completed the questionnaires 6 months after the intervention. No differences were observed between both cohorts, except for the function categorical scale and WOMAC scales, with poorer results in cohort 2 (Table 1).

**Table 1:** Sociodemographic, clinical, and WOMAC preintervention descriptive statistics of samples

Parameter	Cohort 1 (n = 788)	Cohort 2 (n = 445)	*P *value
Age, mean (SD)	69.14 (8.91)	68.42 (9.81)	0.2039
Gender, women	381 (48.35)	221 (49.66)	0.6579
Body mass index			0.2707
< 25	146 (19.36)	99 (23.19)	
25-30	358 (47.48)	198 (46.37)	
≥ 30	250 (33.16)	130 (30.44)	
Surgical risk			0.5047
ASA I-III	773 (98.10)	434 (97.53)	
ASA IV	15 (1.90)	11 (2.47)	
Charlson comorbidity index			0.9341
0	463 (58.76)	266 (59.78)	
1	218 (27.66)	121 (27.19)	
>1	107 (13.58)	58 (13.03)	
Pain categorical scale			0.4593
Minor	32 (4.09)	12 (2.72)	
Moderate	171 (21.87)	96 (21.77)	
Severe	579 (74.04)	333 (75.51)	
Functional limitation categorical scale			0.0076
Minor	79 (10.04)	36 (8.13)	
Moderate	422 (53.62)	206 (46.50)	
Severe	286 (36.34)	201 (45.37)	
WOMAC preintervention domains, mean (SD)			
Pain	54.27 (18.63)	58.16 (19.47)	0.0006
Function	65.19 (16.61)	68.44 (16.85)	0.0011

Data are expressed as frequency (percentage) unless otherwise stated.

Percentages exclude patients with missing data.

The scores for the WOMAC domains range from 0 to 100, with higher scores indicating worse health status.

SD = Standard deviation; ASA = American Society of Anesthesiologists.

### Construct validity

The results of the CFA for the hypothesized model of two latent factors, pain and function, provided satisfactory fit indices in both cohorts (Table 2). The RMSEA values were less than 0.08, and CFI and NNFI values were all exceeding the benchmark of 0.90. All factor loadings were significant (*P*< 0.001) (range, 0.53 - 0.84) and similar in both cohorts, which supported the stability of the subjacent structure of the short questionnaire across the different samples.

**Table 2:** Results of factor loading and fit indexes of Confirmatory Factor Analysis of the WOMAC short questionnaire in both cohorts

Items*	Item description	Cohort 1 (n = 788)		Cohort 2 (n = 445)	
		
		Pain	Function	Pain	Function
Pain item 1	Walking on flat surface	0.75	-	0.77	-
Pain item 2	Up/down stairs	0.84	-	0.84	-
Pain item 4	Sitting or lying	0.53	-	0.59	-
Function item 1	Descending stairs	-	0.74	-	0.74
Function item 2	Ascending stairs	-	0.74	-	0.77
Function item 3	Rising from sitting	-	0.67	-	0.67
Function item 6	Walking on flat surface	-	0.69	-	0.72
Function item 7	Getting in/out of a car	-	0.67	-	0.71
Function item 8	Shopping	-	0.71	-	0.70
Function item 9	Putting on socks	-	0.55	-	0.53
Function item 15	Getting on/off toilet	-	0.66	-	0.67

χ^2 ^(df)		226.11 (40)		119.97 (40)	
RMSEA		0.0792		0.0690	
CFI		0.9539		0.9650	
NNFI		0.9366		0.9518	

*Items are referred to by the original name in the WOMAC long form.

df = degrees of freedom; RMSEA = root mean square error of approximation; CFI = comparative fit index; NNFI = non-normed fit index.

Correlation between the two latent factors (pain and function) is set to be different from 0, therefore both latent factors are specified to be intercorrelated. The estimation of the correlation coefficient was 0.89 in the first cohort and 0.82 in the second one.

Covariance was specified between the error items of the following three pair of items: "Pain walking on flat surface" and "functional limitation walking on flat surface", "pain up/down stairs" and "functional limitation ascending stairs", and "functional limitation getting in/out of a car" and "functional limitation putting on socks".

Regarding the results of the Rasch analyses for the WOMAC pain and function short scales (Table 3), items were separated by 0.10 or more logit unit in both cohorts. Items were equally ranked based on their level of difficulty (*δ*) in both cohorts, which supported the stability of items across the different samples. Unidimensionality was supported with infit and outfit statistics ranging between 0.7 and 1.3, except the item "pain on sitting or lying" relative to pain scale in the first cohort (infit = 1.33, outfit = 1.32) and the item "putting on socks" relative to function scale in cohort 2 (infit = 1.32). Furthermore, the PCA of the residuals did not yield additional factors with eigenvalues exceeding 3, since the second eigenvalue was 1.2 for the pain scale and 1.4 for the function scale in both cohorts, implying that the unidimensionality was not violated. In both cohorts, the person and item separation indexes exceeded 2, indicating reliability over 0.80. The presence of DIF by gender was not detected, given that in no case, the difference in the level of severity according to gender was statistically significant neither it was higher than 0.5 logits.

**Table 3:** Severity levels, standard errors, and goodness of fit indices of the pain and function short scales with application of the Rasch model in both cohorts

Items*	Item description	Cohort 1 (n = 788)					Cohort 2 (n = 445)				
		***δ*** **(logit)**	**SE**	**Infit MNSQ**	**Outfit MNSQ**	**Rank based on logit**	***δ*** **(logit)**	**SE**	**Infit MNSQ**	**Outfit MNSQ**	**Rank based on logit**

Pain^†^											
Item 4	Sitting or lying	2.21	0.07	1.33	1.32	1	2.30	0.09	1.30	1.29	1
Item 1	Walking on flat surface	-0.15	0.07	0.76	0.75	2	-0.07	0.09	0.84	0.87	2
Item 2	Up/down stairs	-2.06	0.07	0.88	0.89	3	-2.23	0.09	0.79	0.79	3

Function^‡^											
Item 6	Walking on flat surface	1.42	0.05	0.88	0.89	1	1.34	0.07	0.87	0.87	1
Item 15	Getting on/off toilet	0.83	0.05	1.15	1.14	2	0.96	0.07	1.16	1.15	2
Item 1	Descending stairs	0.63	0.05	1.01	0.99	3	0.37	0.07	1.00	0.97	3
Item 8	Shopping	0.01	0.06	1.07	1.04	4	0.01	0.08	1.08	1.04	4
Item 3	Rising from sitting	-0.15	0.06	0.93	0.95	5	-0.01	0.08	0.96	1.00	5
Item 2	Ascending stairs	-0.25	0.06	0.86	0.85	6	-0.36	0.08	0.84	0.79	6
Item 7	Getting in/out of car	-0.96	0.06	0.84	0.81	7	-0.91	0.08	0.85	0.82	7
Item 9	Putting on socks	-1.53	0.06	1.30	1.17	8	-1.41	0.09	1.32	1.22	8

*Items are referred to by the original name in the WOMAC long form.

^†^Cohort 1: person separation index = 1.55; item separation index = 24.98; cohort 2: person separation index = 1.56; item separation index = 19.44.

^‡^Cohort 1: person separation index = 2.25; item separation index = 15.39; cohort 2: person separation index = 2.21; item separation index = 10.44.

*δ *= level of severity (higher values indicate higher severity); SE = standard error; MNSQ = mean square fit statistic.

### Reliability

Cronbach's alpha coefficient was 0.74 for the WOMAC pain short scale, and 0.88 for the function short scale, which was superior to the minimum value of 0.70.

### Convergent and discriminant validity

The correlation coefficients between the WOMAC pain and function short scales and the SF-36 domains were all lower than the Cronbach's alpha of the WOMAC-SF scales (Table 4). As hypothesized, the highest correlation coefficient of the WOMAC pain and function short scales were found with the SF-36 bodily pain and physical functioning domains respectively (-0.48 and -0.54).

**Table 4:** Correlation between the WOMAC short scales and SF-36 domains, and known-groups validity of the WOMAC short scales in cohort 1 (n = 788)

	WOMAC short scales	
	
SF-36 domains	Pain ρ coefficient	Function ρ coefficient
Physical functioning	-0.44	-0.54
Role physical	-0.34	-0.36
Bodily pain	-0.48	-0.50
General health	-0.19	-0.17
Vitality	-0.32	-0.33
Social functioning	-0.38	-0.38
Role emotional	-0.17	-0.13
Mental health	-0.29	-0.25
Summary physical component	-0.34	-0.41
Summary mental component	-0.28	-0.25

	**Pain** **Mean(SD)**	**Function** **Mean(SD)**

Pain categorical scale		
Minor (n = 32)^a^	24.74 (12.96)^b, c^	46.23 (17.56)^b, c^
Moderate (n = 171)^b^	43.68 (14.16)^a, c^	57.97 (16.93)^a, c^
Severe (n = 579)^c^	61 (17.16)^a, b^	72.67 (14.99)^a, b^
*P *value	< 0.0001	< 0.0001
Functional limitation categorical scale		
Minor (n = 79)^a^	43.38 (16.63)^b, c^	54.06 (17.48)^b, c^
Moderate (n = 422)^b^	51.70 (17.53)^a, c^	65.38 (15.70)^a, c^
Severe (n = 286)^c^	64.94 (17.56)^a, b^	76.68 (15.43)^a, b^
*P *value	< 0.0001	< 0.0001

ρ: Spearman correlation coefficient.

Data are expressed as the Spearman correlation coefficient when studying the correlation between the WOMAC short scales and the SF-36 domains, and as the mean (SD) when comparing the WOMAC short scales according to the pain and functional limitation short categorical scales.

The scores for the WOMAC domains range from 0 to 100, with higher scores indicating worse health status. The scores for the SF-36 domains range from 0 to 100, with higher scores indicating better health status.

^abc ^Superscript letters indicated differences among the three subgroups by Scheffe's test for multiple comparisons at *P*< 0.05.

### Known-groups validity

The differences in the WOMAC pain and function short mean scales were significant among the three severity groups according to the pain and function categorical scales (Table 4). Patients with a higher level of severity had significantly (*P*< 0.0001) higher scores on the WOMAC pain or function short scale.

### Responsiveness

There were no significant differences among the participants who responded to the follow-up and those who did not. Both the WOMAC pain and function short scales showed minor floor and ceiling effects (< 2%) before the intervention (Table 5). After the intervention, the WOMAC pain and function short scales increased 39.28 and 39.99 points, respectively, both of which were significant (*P*< 0.0001). The SES and SRM responsiveness parameters were much higher than 0.80 in both pain and function short scales, indicating large changes (Table 5).

**Table 5:** Responsiveness parameters 6 months after intervention in the WOMAC short scales in cohort 1 (n = 590)

Parameters	WOMAC short scales	
	
	Pain	Function
% at floor		
Preintervention	0.68	0.17
Postintervention	31.21	6.24
% at ceiling		
Preintervention	1.88	1.89
Postintervention	0.17	0.17
Mean (SD)		
Preintervention	55.69 (18.64)	67.88 (17.44)
Postintervention	16.36 (17.95)	27.74 (19.48)
Change	39.28 (23.14)	39.99 (23.14)
*P *value*	< 0.0001	< 0.0001
SES	2.11	2.29
SRM	1.70	1.73

*Paired *t*-test to compare the mean preintervention and postintervention scores.

% at floor = percentage of the study population at the lowest possible scale level; % at ceiling = percentage of the study population at the highest possible scale level; SD = Standard deviation; SES = Standardised effect size; SRM = Standardised response mean.

The scores for the WOMAC domains range from 0 to 100, with higher scores indicating worse health status.

Changes were calculated by subtracting postintervention scores from preintervention scores; a positive result indicates a gain.

### Agreement between the long and short womac forms

**Figure 1:**
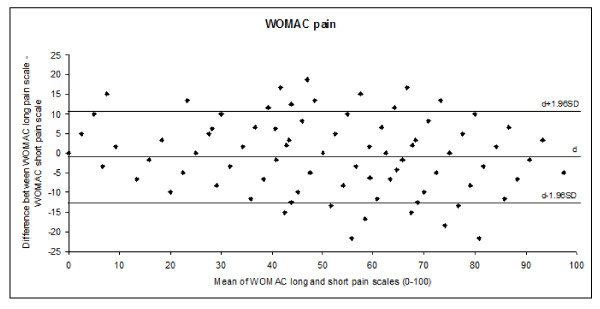
**The Bland-Altman plot shows the difference in the WOMAC long and short pain scales plotted against the mean value of these two scales**. The three horizontal lines indicate the mean individual differences d ± 1.96 SD (limits of agreement). The mean (SD) of the WOMAC long and short pain scales at baseline were 54.27 (18.63) and 55.70 (18.93), respectively. The mean (SD) of the difference between both scales was -1.47 (6.15). Limit of agreement: -13.52 to 10.58.

**Figure 2:**
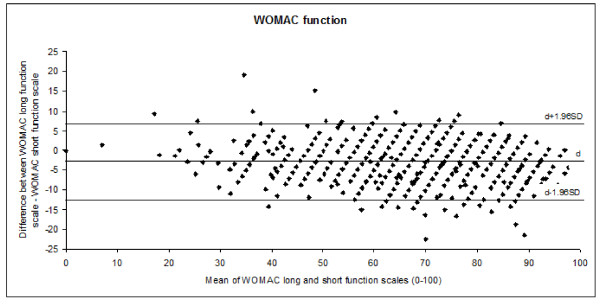
**The Bland-Altman plot shows the difference in the WOMAC long and short function scales plotted against the mean value of these two scales**. The three horizontal lines indicate the mean individual differences d ± 1.96 SD (limits of agreement). The mean (SD) of the WOMAC long and short function scales at baseline were 65.19 (16.61) and 68.36 (17.29), respectively. The mean (SD) of the difference between both scales was -3.15 (4.90). Limit of agreement: -12.75 to 6.45.

The long and short WOMAC scales at baseline, 6 months after the intervention, and the change scores were highly correlated (pain, r = 0.94, 0.97, and 0.94, respectively; and function, r = 0.95, 0.98, and 0.96, respectively). Agreement between the WOMAC long and short scales evaluated by the Bland-Altman approach is shown in Figure 1 and 2. For both domains, more than 95% of the differences between the two scales can be expected to be within the limits of agreement, and the variability was random and uniform along the range of values.

## Discussion

The results of the current prospective study with two independent and large cohorts of patients who underwent THR at different hospitals and who were followed to 6 months support the validity, reliability, and responsiveness of the new 11-item version of the WOMAC. To the best of our knowledge, this is the first study to validate a shortened WOMAC version as a whole tool, including both pain and function dimensions, and most importantly, the first valid, reliable, and responsive WOMAC short version proposed in Spanish.

The WOMAC questionnaire is widely used both in research studies in orthopedic or rheumatologic processes as in clinical practice [1-5,7]. One of the major disadvantages of self-administered questionnaires has been the burden of its completion [41]. In some epidemiological and clinical studies, patients usually have to complete several questionnaires implying a great burden. In clinical practice, where information is collected to evaluate response to treatment, the goal is to involve as little effort as possible for both the patient and the physician. Therefore, if using a shortened version the same information is collected but with little burden, the instrument would be useful. In addition, another disadvantage of self-administered questionnaires has been the low response rate, which greatly affects the study validity [15,16]. Patients missing items has important implications for data collection, completion, and analysis. However, it has been shown that shorter version of the questionnaires would significantly increase the response rate [15], and the compliance increased when the respondent was asked to complete an appreciably smaller set of questions [42]. Therefore, a shorter version would further enhance its applicability in epidemiologic studies, and daily clinical practice [13]. On the other hand, a consequence of the reduction of items is a loss in content validity, the comprehensiveness with which each domain is sampled, and investigators must be cognizant of this issue when they reduce the number of items [12]. Because of a greater length of the questionnaire, it provides a detailed insight of different dimensions. However, this might also be a disadvantage, because of reduced patient compliance and incomplete response [14]. Therefore, it would be very useful to have a shortened WOMAC version in Spanish, which retains the same good psychometric properties of its original version.

The aim of the current study was to propose a new short WOMAC form and validate it in Spanish. Fairclough [43] commented that it is preferable to select a previously validated instrument than to create a new one. Considering this, and according to the different short versions of the WOMAC pain domain proposed by other investigators [19,20], we selected the shortened pain scale proposed by Davis et al [19]. They shortened the WOMAC pain domain using Rasch analysis in a community sample of 773 patients with a hip or knee complaint. The authors concluded that the pain short scale fits the Rasch model and has interval-level scaling properties, and the stability of the model also was supported by a sample of 1,151 surgical patients. Rothenfluh et al. [20] proposed a different three-item pain short version that had two items in common with the version proposed by Davis et al. [19], but the authors based it on a very small sample of patients with hip OA (n = 57). Taking into account our objectives, the methodology used by Davis et al. [19] for the reduction study, the larger sample size, and that both shortened pain domains had the same number of items, we decided that the pain short form proposed by Davis et al. [19] was more adequate.

Regarding the WOMAC function short forms, other versions have been proposed by different authors [11,19-21]. Davis et al. [19], who based their new version on the Rasch model, also proposed a shortened version of the function scale. Nevertheless, they only excluded three items from the original version, and we did not consider short enough. Rothenfluh et al. [20] also proposed a nine-item short version of the function scale based on the Rasch model but used a very small sample of patients with hip OA (n = 57). Given that our target population is composed of patients with hip OA, we did not consider large enough the sample they used. Whitehouse et al. [21] reduced the 17-item function scale to seven items by a clinically driven process based on the opinions of 36 orthopaedic and rheumatology personnel. The authors studied the validity, reliability, and responsiveness of the short scale in patients with hip or knee OA [21], and the criterion validity and repeatability of this reduced function scale also was assessed in a sample of 100 patients, but only 30 had THR [42]. This short function scale also was validated in an independent cohort, but using a sample of patients with knee OA [14]. Finally, Tubach et al. [11], reduced the function scale from 17 items to eight, based on the opinions of 1,362 patients with hip or knee OA and 399 rheumatologists. This short function scale was validated in an independent sample of patients with hip or knee OA, and it was found to be responsive, reproducible, and valid [25]. Although Whitehouse et al. [21] and Tubach et al. [11] used similar methods for shortening the scales, the latter considered more expert opinions, added patient opinions, and the scale was validated by also considering patients with hip OA. Therefore, we selected the function short scale proposed by Tubach et al. [11].

The validation studies of the various shortened WOMAC versions [11,14,19-21,25,42] have consisted of studying the measurement properties of the corresponding shortened WOMAC pain or function scales individually. In our study, we validated our new 11-item WOMAC-SF as an entire tool, including both pain and function dimensions, and studying the construct validity of the short version to test the hypothesis that the 11 items in the questionnaire comprised two separate factors. Validation of the 11-item WOMAC-SF using CFA provides the questionnaire with greater construct validity. The CFA results confirmed the hypothesized internal structure of the two latent factors, given that all fit indices were satisfactory and all factor weights exceeded the recommended thresholds [26-29]. We also confirmed the internal structure of the 11-item WOMAC-SF by CFA performed in an independent cohort. A possible limitation could be the violation of the normal distribution of items when using the CFA. However, it has been argued that the maximum likelihood estimation procedure appear to be fairly robust against moderate violation of this assumption [29]. In addition, some studies, based upon experience or computer simulations, have claimed that scales with as few as five points yield stable factors [37]. Therefore, taking into account that we use a 5-points Likert scale, a maximum likelihood estimator procedure, and that we have a large sample size, with practically equal results in both cohorts, we think that our CFA results are reliable and stable.

The Rasch method applied to the three-item pain short domain and the eight-item function short domain provided adjustment levels (infit and outfit) and unidimensionality sufficient to be considered adequate, providing major evidence of construct validity. Although two of the items, the item "pain on sitting or lying" relative to pain scale and the item "putting on socks" relative to function scale, presented infit or outfit statistics slightly above the recommended threshold of 1.3, taking into account the satisfactory results obtained from the rest of analysis, such as PCA of the residuals, the functioning of the rating scale categories, the absence of DIF by gender in both items, and the item and person separation indexes, we do not consider that the slight difference in these infit or outfit indexes with respect to the recommended limit 1.3 is large enough to conclude that these two items are misfitting items. Regarding the three-item pain short form, the results were similar to those reported by Davis et al [19]. Considering that the criteria were satisfactory, we concluded that the shortened WOMAC pain scale fit the Rasch model. Regarding the eight-item function short form, we obtained a scale that shows the fundamental properties of model fit and unidimensionality.

Analysis of the internal consistency allowed us to confirm the hypothesis that the items that comprised the pain short scale or those that comprised the function short scale measured the same concept as Cronbach's alpha coefficient exceeded the threshold of 0.70 [36]. For the function short scale, the results were similar to or slightly higher than those reported by the original authors of the short form [11,25]. Further, the reliability of the 11-item WOMAC-SF, although it was as high as that for the original Spanish WOMAC questionnaire (0.82 for pain domain and 0.93 for function domain) because of the reduction of the number of items, it was slightly lower, indicating that it maintained excellent internal consistency [8].

The convergent and discriminant validity of the WOMAC-SF was assessed by examining the relationship between the pain and function short scales and the factors of the SF-36. Validity was demonstrated by correlation coefficients lower than the internal consistency of the short forms and by confirming the hypothesis that the highest correlation coefficients were found between the WOMAC pain short form and the SF-36 bodily pain domain and between the WOMAC function short form and the physical function domain of the SF-36. Baron et al. [25] also reported satisfactory convergent validity of the eight-item function WOMAC short form, but they used measures other than the SF-36. Whitehouse et al. [21] studied the convergent validity of their proposed seven-item function short form using the SF-36 physical function domain, and although the results were similar to those we obtained, in our case the correlation coefficient was slightly higher. Further, we obtained similar results to those of the original WOMAC questionnaire [8], since they also found the highest correlation coefficient between the WOMAC pain and function long scales and the SF-36 bodily pain and physical functioning domains (-0.55 and -0.59, respectively). Otherwise, the WOMAC-SF maintained excellent known-groups validity similar to that of the original WOMAC questionnaire [8], since they also observed that the more severity level, the higher their WOMAC pain and function long scores were.

The 11-item WOMAC-SF showed good responsiveness 6 months after the intervention. Responsiveness parameters were substantially above the 0.80 threshold for designating large change [39]. Tubach et al. [11] and Baron et al. [25] also reported this finding for the function short form, although we found much higher responsiveness parameters, probably due to the follow-up period. We considered a follow-up of 6 months, whereas they considered 4 weeks. Whitehouse et al. [21], who purposed a seven-item function WOMAC-SF, studied the responsiveness considering follow-up periods of 3 months and 1 year, and Auw Yang et al. [14], who validated the previous seven-item function WOMAC-SF in a different cohort, also studied the responsiveness considering follow-up periods of 3 and 6 months. Nevertheless, the responsiveness parameters of the seven-item function WOMAC-SF that they reported [14,21] were much lower than our responsiveness parameters of the eight-item function short form that we proposed, indicating that the eight-item function short form is more responsive than the seven-item function short form proposed by Whitehouse et al. [21]. Further, the responsiveness results of the 11-item WOMAC-SF we obtained were similar to those of the original WOMAC questionnaire [9], given that they also found minor floor and ceiling effects (< 2%) before the intervention, and the SES and SRM responsiveness parameters were practically equal (2.10 and 1.86 respectively for pain domain, and 2.34 and 1.80 respectively for function domain).

The strong correlation between the long and short WOMAC pain or function scales and the high agreement in scores examined by the Bland-Altman approach [40] support the hypothesis that the shortened scale captures pain and functional status as well as the original WOMAC version. Our results are similar to those found by Tubach et al. [11] and Baron et al. [25].

A possible limitation of the current study was the use of the data provided by the original WOMAC long form to validate the 11-item WOMAC-SF [25]. This might constitute a framing bias and lead to overestimation of the similarity between the two forms [21,25]. Although this problem is inherent in many validation studies [11,25], in the current study, whenever possible, we analyzed separate samples to compensate for this problem as much as possible. Nevertheless, the 11-item WOMAC-SF must be validated in a new independent sample of patients with hip OA and in different languages. Besides, the original WOMAC has been used in patients with hip or knee OA, consequently this 11-items short form could probably be applicable in both patients with hip or knee OA. However, we have based our study only on patients undergoing total hip replacement, and therefore, further validation studies in patients with different arthroplasties would be necessary to be completely sure about the applicability of this short WOMAC form.

In addition, an instrument must be reliable, valid, and responsive to be useful. Although we studied the reliability of the 11-item WOMAC-SF by means of the Cronbach alpha coefficient to measure the internal consistency, the reliability study should be complemented with a test-retest study. Regarding responsiveness, missing data are a key limitation of the prospective cohort design and a usual finding when conducting follow-up studies [11,21,25]. In our case, there was a very good response rate before the intervention (about 80%) in both cohorts, and 6 months after it (about 75%). These losses occurred despite our mailing up to two reminders and contacting nonresponders by telephone. However, no differences were observed in relevant variables when responders were compared with nonresponders. Therefore, although a bias may have been present in our responsiveness study due to missing data, it is likely to be minor and we believe the results are generalizable to the entire sample.

## Conclusions

In conclusion, we proposed an 11-item WOMAC-SF, based on previous studies, for patients with hip OA undergoing THR. This complete validation process, which used two independent and large patient samples and combined classical and contemporary methods, such as Rasch analysis, showed that the 11-item Spanish WOMAC-SF is valid, reliable, and responsive for measuring pain and function in patients with hip OA undergoing THR, and most importantly, the first WOMAC short version proposed in Spanish. Its simplicity and easy of application will increase its acceptability and usefulness within the orthopaedic community, and, therefore, it may be of interest in routine practice given that the goal is to collect information involving as little effort as possible for both the patient and the physician. In clinical research, where patients usually have to complete several questionnaires implying a great burden, short questionnaires result in improved patient compliance and response rates, therefore this shorter version will further enhance its applicability. In conclusion, this short version is a good alternative to the original WOMAC questionnaire, since the 11-item WOMAC-SF retains properties of the original WOMAC version.

## List Of Abreviations

ASA: American Society of Anesthesiologists; CFA: Confirmatory factor analysis; CFI: Comparative fit index; DIF: Differential item functioning; MNSQ: Mean square fit statistic; NNFI: Non-normed fit index; OA: Osteoarthritis; PCA: Principal components analysis; RMSEA: Root mean square error of approximation; SD: Standard deviation; SE: Standard error; SES: Standardized effect size; SF-36: Short Form-36; SRM: Standardized response mean; THR: Total hip replacement; WOMAC: Western Ontario and McMaster Universities Osteoarthritis Index; WOMAC-SF: Western Ontario and McMaster Universities Osteoarthritis Index short form.

## Competing interests

The authors declare that they have no competing interests.

Financial Support: Supported in part by grants from the Fondo de Investigación Sanitaria (98/001-01 to 03; 01/0184).

## Authors' contributions

AB has participated in the conception and design of the study, in the analysis and interpretation of data, and has been involved in drafting the manuscript; JMQ and AE have participated in the conception, design and coordination of the study, have helped to draft the manuscript and have been involved in revising it critically for important content; CLH and MO have made substantial contribution to acquisition of data, and have helped to draft the manuscript. All authors have read and approved the final manuscript.
